# Successful management of in-transit cutaneous squamous cell carcinoma with anti-PD-1 immunotherapy

**DOI:** 10.1186/s12957-023-03179-3

**Published:** 2023-09-15

**Authors:** Chris Furkert, Richard Martin, Avinash Sharma, Gareth Rivalland

**Affiliations:** 1 North Shore Hospital, Auckland, New Zealand; 2 Auckland City Hospital, Auckland, New Zealand

**Keywords:** Squamous cell carcinoma, In-transit, In-transit metastases, Immunotherapy, Anti-PD-1

## Abstract

This report describes the case of a 70-year-old man with metastatic squamous cell carcinoma (SCC) of the right lower leg. Soon after definitive surgical management of the primary and nodal metastases with curative intent, he relapsed, developing aggressive in-transit metastatic disease and recurrent nodal disease. The patient was treated with systemic immunotherapy alone, which not only prompted the progressive nodal metastases to regress, but also resulted in a complete response of the in-transit disease. This situation is previously undescribed in the medical literature.

## Introduction

Squamous cell carcinoma is an extremely common form of cutaneous malignancy, and Auckland, New Zealand has the highest reported incidence of invasive non-melanoma skin cancers in the world [1]. Although most cutaneous squamous cell carcinomas (cSCC) will be treated with local therapy, this form of malignancy has a documented rate of metastasis of up to 5% [2]. The CARSKIN and KEYNOTE-629 studies established the role of pembrolizumab, an anti-PD-1 humanised monoclonal antibody, in the treatment of recurrent or metastatic squamous cell carcinoma (SCC), leading to approval by the US Food and Drug Administration in June 2020 [3, 4]. In the KEYNOTE 629 trial, the objective response rate (ORR) was 35% in locally advanced or metastatic cSCC, with 10.5% complete responses (CR). Unfortunately, this indication remains publically unfunded in New Zealand, [5] though the medication is accessible for metastatic cSCC in the private sector [6]. Less well documented is the use of this medication for the treatment of in-transit metastases, in which tumour deposits occur in either the skin or subcutaneous tissue between the site of the primary lesion and the relevant nodal basin, which in themselves are a rare manifestation of cSCC [7, 8]. In this report we will discuss a case in which known in-transit disease responded completely to the systemic use of anti-PD-1 immunotherapy.

## Case

Patient DM presented in June 2021 as a 70-year-old male with a 7 cm right lower leg lesion, clinically in keeping with cSCC, and palpable lymphadenopathy in the right inguinal nodal basin. He was otherwise fit at the time, an ex-smoker with a background of hypertension and hyperlipidaemia, who still taught martial arts. At the time of his initial workup, he was noted to have a markedly elevated prostate-specific antigen (PSA), which led to the concurrent diagnosis of International Society of Urological Pathologists (ISUP) Grade 5 prostate cancer on transrectal ultrasound (TRUS) biopsy. A fine needle aspiration (FNA) of the inguinal lymph nodes, however, confirmed metastatic cSCC to the superficial groin. A staging computed tomography (CT) demonstrated no evidence of more distant spread, and magnetic resonance imaging (MRI) of the right leg showed a clear margin over the tibial periosteum. Pre-operative imaging also demonstrated a right leg deep vein thrombosis (DVT), and he was commenced on low weight molecular heparin. Despite his excellent premorbid status, by the time he came forward for surgical intervention, pain in the right leg had rendered DM largely immobile and wheelchair dependent.

He was brought forward for wide local excision of the primary lesion from the right lower leg in August 2021, at which time the tibial periosteum was taken en bloc with the specimen and the outer table of the bone removed with a burr. The defect was covered with a split skin graft (SSG), and a right inguinal superficial groin dissection was concurrently performed. The histology from this operation returned a 85 × 68-mm SCC, Breslow 10 mm, Clark’s Level 5, with 8-mm radial and 0.6-mm deep margins. Metastatic carcinoma was shown in 4 of 10 nodes, with some minimal extranodal extension (pT2, pN2b, pMx). Unfortunately, the groin wound edges subsequently deteriorated and became necrotic. A sartorial switch was performed in September, with the intention to allow the application of a negative pressure dressing to the area to expedite healing and facilitate local radiotherapy. After a period of negative pressure treatment, a further SSG was used to cover the residual defect in the right groin.

**Fig. 1:**
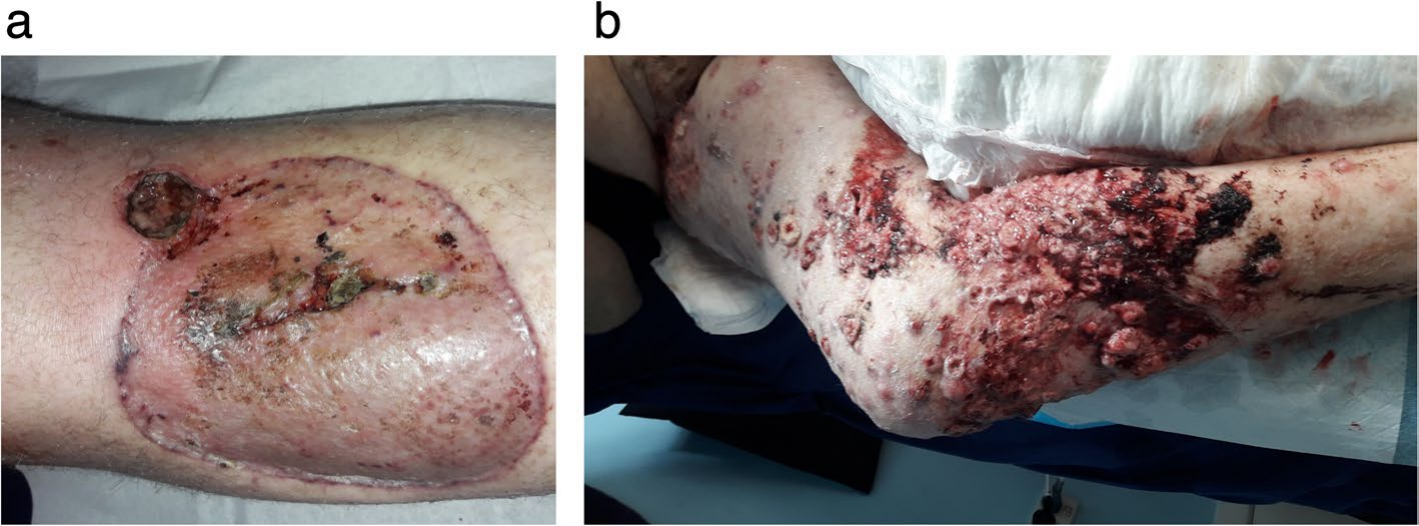
**a** Right leg showing the site of recently excised in-transit metastasis, adjacent to the primary site Jan 13, 2022. **b** Right leg, showing the progression of in-transit disease, Feb 2, 2022

Based on his histology, adjuvant radiation was recommended to treat the primary resection site and the groin after wound healing. However, this never eventuated. In October 2021, DM developed a new cutaneous lesion immediately superior to his primary site. Excision biopsy of this confirmed metastatic SCC (Fig. 1a). Further in-transit lesions began to rapidly develop in the left thigh and adjacent to the primary lesion site, as well as new palpable nodularity within the right groin (Fig. 1b). These findings were corroborated on staging CT, with significant nodal progression along the right external iliac vessels. He was also diagnosed with recurrent DVT, resulting from the physical compression of these vessels by a large necrotic nodal mass.

**Fig. 2:**
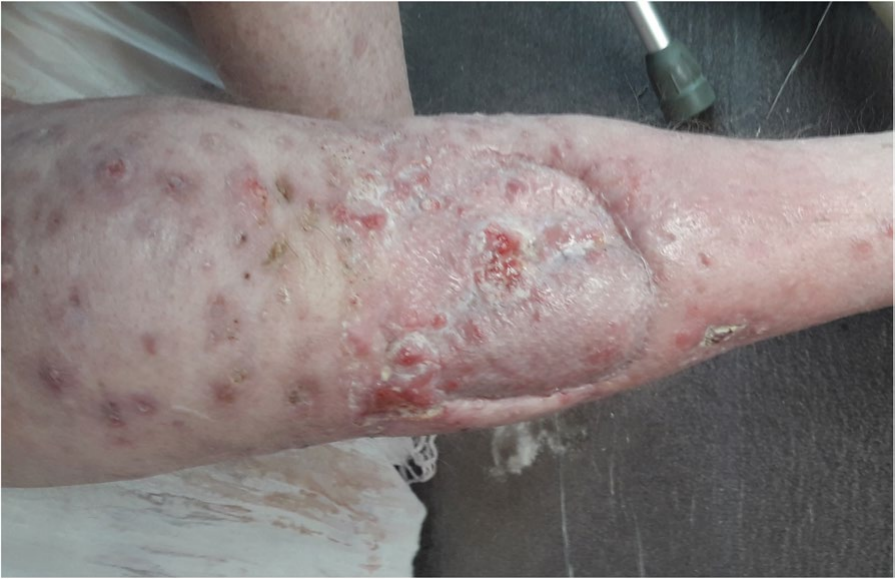
Right leg showing regression of in-transit disease following treatment with pembrolizumab, Mar 21, 2022

Due to the aggressive metastatic recurrence and biology of his malignancy, he was referred to the private sector for consideration of urgent treatment with immunotherapy. Immunohistochemistry for PD-L1 was performed, and showed 2 + /3 + staining in 80% of tumour cells, with the control tissue appropriately stained. This test was performed using PD-L1 (22C3 pharmDX Mouse Monoclonal) immunoperoxidase histochemistry on the Dako Autostainer Link 48 with EnVision FLEX detection kit. He commenced self-funded pembrolizumab therapy on Jan 27, 2022, with an excellent documented response. Pembrolizumab was given at a flat dose of 200 mg once every 3 weeks via intravenous infusion. Not only has he had interval improvement of his lymphadenopathy documented over the course of several CTs, but his in-transit nodes have also completely regressed (Fig. 2). This radiological change also corresponded with a significant clinical improvement in his daily function. He is no longer reliant on his wheelchair, and is in fact mobilising short distances with crutches, though he still requires assistance with activities of daily living. DM did not experience any immune-related adverse events, though he did experience grade 1 episodes of gastritis, left inguinal cellulitis and urinary tract infection. These symptoms settled with standard therapy and did not require immunosuppression.

## Discussion

This case is unique in the literature in describing the clinical and radiological response of in-transit squamous cell carcinoma exclusively to systemic anti-PD-1 immunotherapy. From pre-clinical studies, cSCC should theoretically be sensitive to checkpoint inhibitor therapy, as they commonly demonstrate PD-1 protein expression and high tumour mutation burden due to UV radiation-induced damage to DNA [9]. A case snapshot has been published detailing a partial response of in-transit squamous cell carcinoma to immunotherapy with cemiplimab and concurrent radiotherapy; [10] but this is the first documented case with response to systemic immunotherapy alone. Historically, treatment of in-transit disease has been limited to recurrent excisions, focal radiotherapy, or in some instances isolated limb infusion of traditional chemotherapy agents [11]. Whilst these methods have been shown to be partially effective, our case demonstrates that in-transit cSCC disease recurrence can be treated with systemic immunotherapy alone. In this case, we also observed simultaneous treatment effects in both the in-transit disease and more distant metastatic disease. Unfortunately, pembrolizumab is one of only limited immune checkpoint inhibitor monoclonal antibodies available in any form in New Zealand, and its’ publicly funded indication remains limited to patients with metastatic and unresectable melanoma. It is our hope that the benefit from this medication derived by patients with metastatic squamous cell carcinoma of any form encourages the New Zealand regulatory bodies to consider subsidising this medication for appropriate patients in the future. The morbidity and expense involved with recurrent operative intervention would be worth exploring in a separate audit, which could be then compared against the anticipated expenditure and benefit from immunotherapy.

## Data Availability

Not applicable.

## References

[1] Pondicherry A, Martin R, Meredith I, Rolfe J, Emanuel P, Elwood M (2018). The burden of non-melanoma skin cancers in Auckland New Zealand. Aust J Dermatol.

[2] Caudill J, Thomas JE, Burkhart CG. The risk of metastases from squamous cell carcinoma of the skin. Int J Dermatol. 2023;62(4):483–6. 10.1111/ijd.16164 35324009

[3] Maubec E, Petrow P, Scheer-Senyarich I, Duvillard P, Lacroix L, Gelly J (2011). Phase II study of cetuximab as first-line single-drug therapy in patients with unresectable squamous cell carcinoma of the skin. J Clin Oncol.

[4] Grob J-J, Gonzalez R, Basset-Seguin N, Vornicova O, Schachter J, Joshi A (2020). Pembrolizumab monotherapy for recurrent or metastatic cutaneous squamous cell carcinoma: a single-arm phase II trial (KEYNOTE-629). J Clin Oncol.

[5] pembrolizumab - New Zealand Formulary [Internet]. Available from: https://nzf.org.nz/nzf_70469. [cited 2022 Nov 16].

[6] Cancer Immunotherapy in New Zealand | Fight Cancer NZ [Internet]. Available from: https://www.fightcancer.co.nz/. [cited 2022 Nov 16].

[7] Ma JHY, Wu A, Veness M, Estall V, Hong A, Borg M (2016). In-transit metastasis from squamous cell carcinoma. Dermatol Surg.

[8] Stewart JR, Ahn JW, Brewer JD (2022). In-transit metastasis of cutaneous squamous cell carcinoma with lymphovascular invasion in an immunocompetent patient. Cureus.

[9] Pickering CR, Zhou JH, Lee JJ, Drummond JA, Peng SA, Saade RE (2014). Mutational landscape of aggressive cutaneous squamous cell carcinoma. Clin Cancer Res.

[10] McLean LS, Rischin D (2022). In-transit cutaneous squamous cell carcinoma. Med J Aust.

[11] Belgrano V, Ben-Shabat I, Bergh P, Olofsson BR (2016). Isolated limb perfusion as a treatment option for rare types of tumours. Int J Hyperthermia.

